# Tetralogy of Fallot: morphological variations and implications for surgical repair

**DOI:** 10.1093/ejcts/ezy474

**Published:** 2019-01-16

**Authors:** Saad M Khan, Nigel E Drury, John Stickley, David J Barron, William J Brawn, Timothy J Jones, Robert H Anderson, Adrian Crucean

**Affiliations:** 1 Department of Paediatric Cardiac Surgery, Birmingham Children's Hospital, Birmingham, UK; 2 Institute of Clinical Sciences, University of Birmingham, Birmingham, UK; 3 Institute of Cardiovascular Sciences, University of Birmingham, Birmingham, UK

**Keywords:** Tetralogy of Fallot, Morphology, Coronary arteries, Aortic override, Cardiac surgery

## Abstract

**OBJECTIVES:**

Tetralogy of Fallot is characterized by anterocephalad deviation of the outlet septum, along with abnormal septoparietal trabeculations, which lead to subpulmonary infundibular stenosis. Archives of retained hearts are an important resource for improving our understanding of congenital heart defects and their morphological variability. This study aims to define variations in aortic override, coronary arterial patterns and ventricular septal defects in tetralogy of Fallot as observed in a morphological archive, highlighting implications for surgical management.

**METHODS:**

The Birmingham Children’s Hospital archive contains 211 hearts with tetralogy of Fallot, of which 164 were analysed [69 (42.1%) unrepaired and 95 (57.9%) operated specimens]. A detailed morphological and geometric analysis was performed using a rigorous 5-layer review process.

**RESULTS:**

Anomalies were observed in the orifices, origins and course of the coronary arteries: 20 hearts (13.0%) had more than 2 orifices and 3 hearts (1.9%) had a single orifice. In 7 hearts (4.3%), a coronary artery crossed the right ventricular outflow tract. The extent of aortic override ranged from 31.0% to 100% (median of 59.5%). The ventricular septal defect was most often perimembranous (139, 84.8%), but we also found muscular (14, 8.5%), atrioventricular (7, 4.3%) and doubly committed juxta-arterial (2, 1.2%) variants.

**CONCLUSIONS:**

Anatomical variations are common and can impact surgical management. Anomalous coronary arteries may require a conduit rather than a transannular patch. Variability in aortic override determines the size of patch used to baffle blood to the aorta. The type of ventricular septal defect affects patch closure and the risk of postoperative conduction defects.

**Figure:**
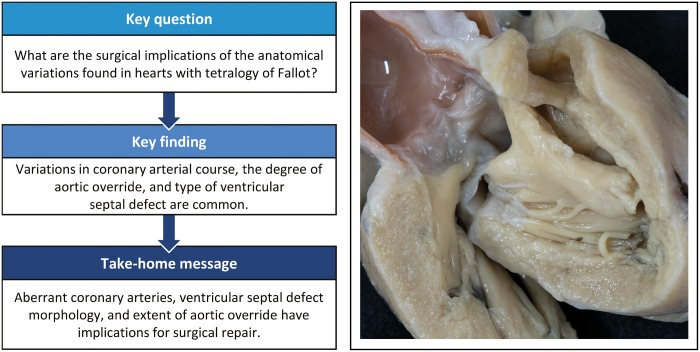

## INTRODUCTION

It is 130 years since Fallot described the 4 cardinal features of *la maladie bleue* [1] namely the complex of pulmonary infundibular stenosis, an interventricular communication, biventricular connection of the aorta and right ventricular hypertrophy. Our understanding of the developmental anomaly, phenotypic features and surgical management has advanced considerably in the intervening years [2–4]. The anterocephalad deviation of the outlet septum, with associated abnormal septoparietal trabeculations, is now accepted as the hallmark of tetralogy [5, 6]. This outlet septal deviation also results in varying degrees of aortic override and can be associated with several types of ventricular septal defect [7].

The combination of lesions can be found with a wide spectrum of associated anomalies, including so-called absent pulmonary valve, pulmonary atresia, atrioventricular septal defect, abnormal branching of the coronary arteries, a right aortic arch and persistence of the left superior caval vein [4, 8, 9]. While some of these structural anomalies may simply be benign variations, others have significant implications for surgical management. Preoperative imaging, therefore, should be focused on variants known to be of significance, such as a coronary artery traversing the right ventricular outflow tract [10]. Advanced knowledge of these factors, and their clinical implications, will inform the discussion with parents regarding prognosis and reintervention and aid planning of the operative repair.

Post-mortem heart specimens stored in historical archives provide a valuable resource for direct examination of the anatomy of such congenital heart defects and their associated anomalies. The transformation of surgical interventions and outcomes over the last 60 years, along with changes in societal attitudes towards post-mortem, highlight the irreplaceability of these collections and their ongoing value for morphological research. The Birmingham Children’s Hospital cardiac archive provides us with the unique opportunity to examine a range of unrepaired and historically operated hearts. Using this archive, we have sought to determine the morphological and geometric features of tetralogy, defining variations such as aortic override, coronary arterial patterns and the morphology of ventricular septal defects which may have implications for surgical management.

## MATERIALS AND METHODS

The Birmingham Children’s Hospital cardiac archive consists of approximately 2000 hearts obtained between 1939 and 2001 that were examined post-mortem and contemporaneously dissected, fixed and stored in 10% formalin [11]. In recent years, the archive was externally audited, a governance framework established and retained hearts extensively catalogued using sequential segmental analysis to facilitate research and teaching [12]. An ethical approval for this study was granted by the Birmingham Children’s Hospital Cardiac Archive committee, including independent members, which oversees the preservation of the collection.

### Specimen selection

We reviewed all specimens in the archive with the phenotypical features of tetralogy of Fallot, specifically anterocephalad deviation of the outlet septum with abnormal septoparietal trabeculations producing subpulmonary infundibular obstruction in the setting of a ventricular septal defect and aortic override. Specimens with pulmonary atresia or so-called ‘absent’ pulmonary valve were excluded. All hearts included had usual atrial arrangement and concordant atrioventricular connections. The ventriculo-arterial connections showed a spectrum between concordant and double-outlet variants depending on the extent of aortic override, but all specimens were confirmed to have aortomitral fibrous continuity. Specimens were classified as unrepaired or operated. An unrepaired heart was defined as one in which no surgical operation had been performed on the myocardium during life; hearts from patients who had undergone only a palliative extracardiac shunt were thereby classified as unrepaired.

### Heart evaluation

**Figure 1:**
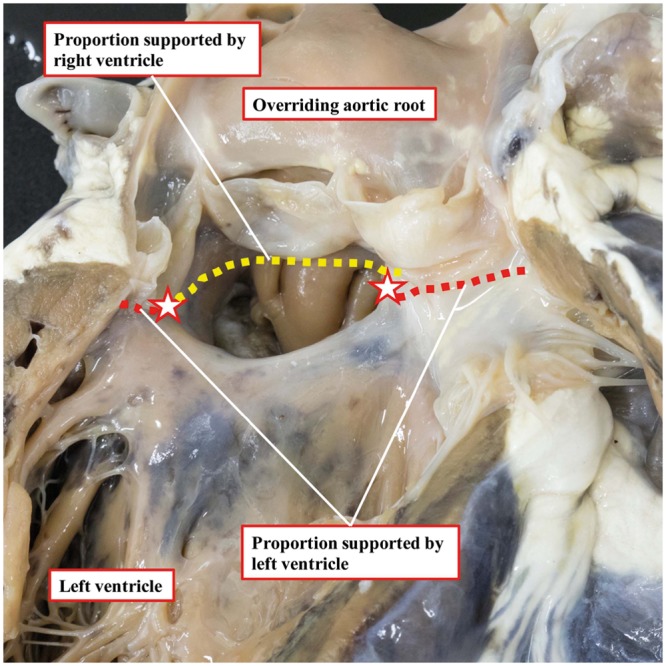
Linear method for the measurement of aortic override, as viewed from the left ventricle. The crest of the muscular ventricular septum was taken as the boundary for the aortic leaflets supported by the ventricular structures (red stars). The proportion of the circumference of the aortic root supported by the left ventricle is the sum of the red dashed lines and that supported by the right ventricle is the yellow dashed line.

We followed a systematic approach, whereby each heart was inspected and measured externally followed by internal examination (S.M.K.) according to a predefined protocol (Supplementary Material, Table S1). The degree of aortic override was measured using 3 independent techniques. Firstly, the ‘leaflet proportion method’, adapted from Winn *et al.* [13], involved dividing each aortic valve leaflet into quarters and measuring the proportion of the aortic valve leaflets lying in the right ventricular cavity. Secondly, a ‘qualitative method’ for assessment of the percentage of aortic override involved visual inspection of the specimen by trained (A.C.) and expert (R.H.A.) morphologists. Thirdly, we introduce a novel ‘linear method’ to measure the percentage of aorta override based on the proportion of the circumference of the aortic root relative to the tangent produced by the ventricular septal crest that is supported by left ventricular structures (Fig. 1). As long as the majority of the circumference of the root is supported by left ventricular structures, the aorta is deemed to be committed to the left ventricle [14]. The left ventricular component was measured because of the way most hearts were dissected, with the tricuspid valve obstructing our view of the aortic root when assessing from the right ventricle. We compared the variability of these 3 techniques for quantifying aortic overriding, as there is currently no consensus in the literature on how best to measure this parameter.

### Review process

All included hearts were assessed according to a rigorous 5-layer review process. Each specimen was independently examined for the study according to the review protocol without knowledge of previous assessments (S.M.K., layer 1). This analysis was compared with previous independent assessments made by in-house (A.C.) and visiting (R.H.A.) expert morphologists (layer 2), along with the observations obtained from the original hospital post-mortem report for each patient (layer 3). The assessor then re-evaluated each heart in light of any additional or conflicting information (layer 4), with any differences being rediscussed (with A.C., layer 5), and resolved by consensus.

This standardized review process was instituted to ensure consistency of the observations and measurements of each specimen. There is currently no reference standard for the assessment of heart specimens. Previous descriptions have been based solely on individual expert opinion. Such an approach introduces chances of error when dealing with morphological descriptions and quantitative measurements. Hence, we implemented our meticulous approach to ensure the validity of our findings.

### Statistical analysis

Statistical analyses were performed using *R* (https://www.r-project.org/). Categorical data are expressed as counts and percentages and continuous variables as medians and interquartile ranges, unless otherwise specified. Bland–Altman plots and histograms of difference were used to compare the 3 methods of measuring aortic override.

## RESULTS

We identified 211 hearts within the archive having tetralogy as currently defined. After exclusions, 164 (77.7%) specimens were retained for the current study, of which 69 (42.1%) were classified as unrepaired, whereas 95 (57.9%) had undergone intracardiac surgical procedures. The retained hearts had been added to the collection between 1939 and 1994 and weighed from 3.80 g to 319.80 g [median 61.1 g; interquartile range (IQR) 26.4–104.6]. Donors were mostly male (88, 58.3%) and ranged from birth to 17 years at the time of death.

### Coronary arteries

**Figure 2:**
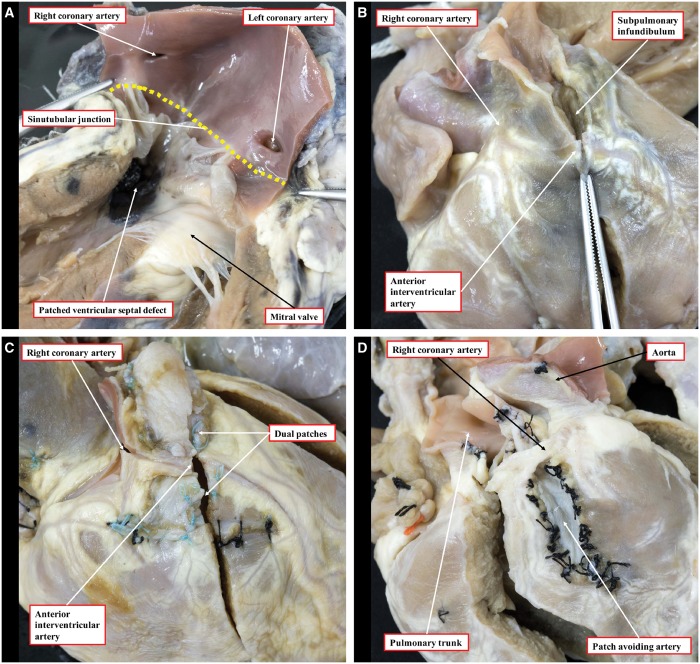
Variation in the orifice position and the course of anomalous coronary arteries crossing the right ventricular outflow tract. (**A**) The high location of the right and left coronary orifices above the sinutubular junction (yellow dashed line). (**B**) An anterior interventricular artery originating from the right coronary artery and coursing towards the apex. (**C**) An operated heart, in which the anterior interventricular artery has been dissected, again originating from the right coronary artery, repaired using the 2-patch technique. (**D**) The aortic root lies to the left of the pulmonary trunk and the right coronary artery crosses the right ventricular outflow tract to achieve its anticipated position in the right atrioventricular groove; a patch has been placed beneath the right coronary artery to avoid incising it.

Multiple anomalies were observed in the orifices, origin and course of the coronary arteries, as shown in Table 1. The aortic root was accessible in 154 hearts, and while most of these specimens had 2 coronary arterial orifices (131, 85.1%), 20 (13.0%) hearts had more than 2 orifices, whereas 3 (1.9%) had a single coronary orifice. Of the overall number, 61 orifices originated at or above the sinutubular junction, with 49 of these positioned at or above the junction of the left coronary sinus and 12 from above the right coronary sinus (Fig. 2). In 7 (4.3%) hearts, a coronary artery was seen to cross the right ventricular outflow tract; this was the anterior interventricular artery in 6 (3.7%) and the right coronary artery in 1 (0.6%) (Fig. 2).

**Table 1:** Variation in the number of coronary arterial orifices, position and anomalous coronary arteries seen crossing the right ventricular outflow tract

Anatomical variant	*n* (%)
Number of coronary orifices	154
1	3 (1.9)
2	131 (85.1)
3	19 (12.3)
4	1 (0.6)
High coronary orifice position	154
Left coronary orifice at STJ	21 (13.6)
Right coronary orifice at STJ	0
Left coronary orifice above STJ	28 (18.2)
Right coronary orifice above STJ	12 (7.8)
Anomalous coronary artery crossing RVOT	164
Anterior interventricular artery	6 (3.7)
Right coronary artery	1 (0.6)

RVOT: right ventricular outflow tract; STJ: sinutubular junction.

### Relative position of the great arteries

**Figure 3:**
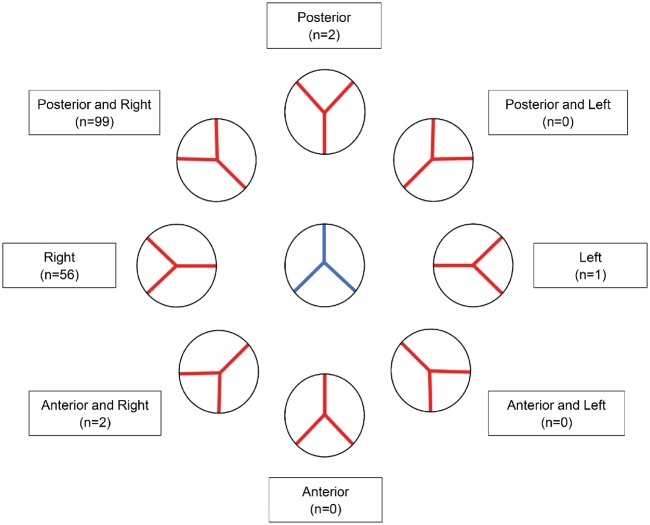
Variation in the position of the aortic root (red) with reference to the pulmonary root (blue).

The position of the aortic valve in relation to the pulmonary valve is illustrated in Fig. 3. In most (99, 61.9%) hearts, the aortic valve was located posterior and to the right of the pulmonary valve. The aortic valve was positioned side-by-side to the right of the pulmonary valve in 56 (35.0%) hearts, anterior and to the right in 2 (1.3%), directly posterior in 2 (1.3%) and side-by-side to the left in 1 (0.6%).

### Aortic override

**Figure 4:**
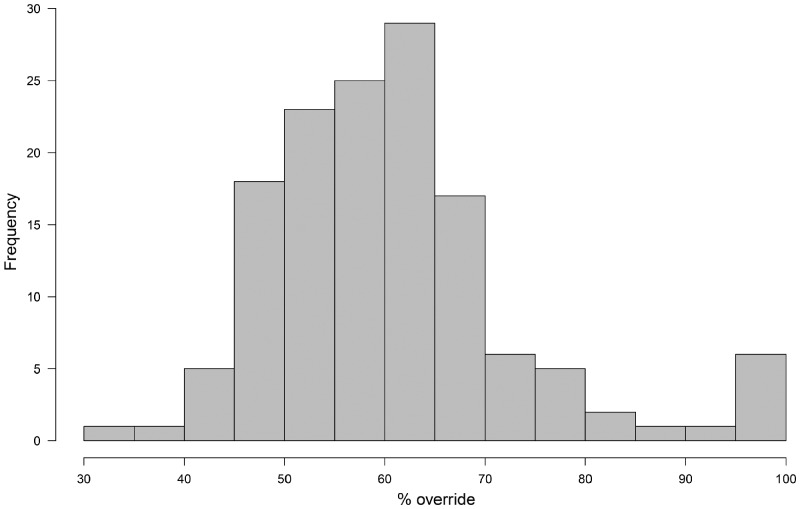
Histogram demonstrating the range of aortic override using the linear method.

**Figure 5:**
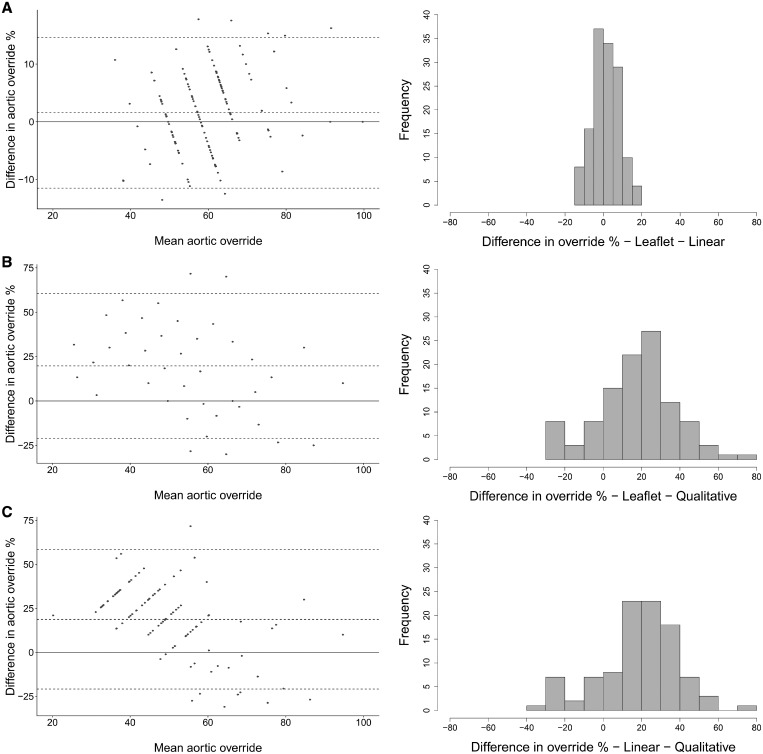
Bland–Altman plots and histograms of difference for measurements of the degree of aortic override comparing (**A**) leaflet proportion versus linear methods, (**B**) leaflet proportion versus qualitative methods and (**C**) linear versus qualitative methods.

The extent of aortic override of the right ventricle ranged from 31.0% to 100% (median 59.5%, IQR 52.7–65.5) using our linear method (Fig. 4) and 33.3% to 100% (median 58.3%, IQR 50.0–66.7) with the leaflet proportion method. Comparisons of the 3 methods for determining aortic override are shown in Fig. 5. There was no difference between the linear and leaflet proportion methods (*P *=* *0.22), but the qualitative method significantly underestimated the degree of override compared with both the linear and leaflet proportion methods (both *P < *0.001) and had much greater variability.

### Ventricular septal defects

**Figure 6:**
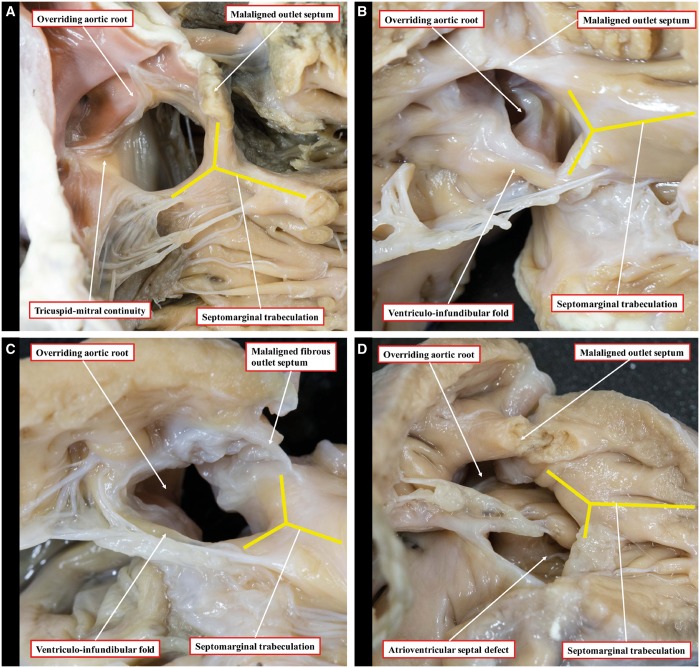
Morphology of the various types of ventricular septal defect, as seen from the right ventricle: perimembranous (**A**), muscular posteroinferior rim (**B**), doubly committed and juxta-arterial (**C**) and atrioventricular septal defect (**D**). The yellow lines show the septomarginal trabeculation.

The differing morphology of the ventricular septal defects is illustrated in Fig. 6. Most defects were perimembranous (139, 84.8%), with 3 (2.2%) of these accompanied by an additional muscular ventricular septal defect. In 14 (8.5%) hearts, there was an isolated muscular outlet defect, produced by a muscular posteroinferior rim interposing between the leaflets of the aortic and tricuspid valves. Of the remaining hearts, in 7 (4.3%), the defect was the ventricular component of an atrioventricular septal defect, whereas in 2 (1.2%), the defect was doubly committed and juxta-arterial, due to fibrous continuity between the leaflets of the aortic and pulmonary valves.

## DISCUSSION

In this study, we have evaluated the morphological variation of human post-mortem hearts with tetralogy of Fallot contained in a large cardiac archive. We found a spectrum of morphological variants and were able to directly quantify parameters that have implications for operative repair. These related in particular to the coronary arteries, the position of the great arteries, the degree of aortic override and the type of ventricular septal defect.

### Coronary arterial abnormalities

We observed variations in the orifices, origin and course of the coronary arteries that may have significant implications for management. A coronary artery crossed the right ventricular outflow tract in almost one-twentieth of the hearts, most commonly the anterior interventricular artery either branching directly off the right coronary artery or arising from its own orifice in the right coronary sinus. These findings are similar to reported clinical series [15, 16]. Anomalous coronary arteries are at risk of transection during complete repair, especially if mistaken for a large infundibular or conal branch [17]. There is no consensus on how to deal with such anomalous coronary arteries. Numerous techniques have been reported, including placement of a right ventricular-to-pulmonary arterial conduit, a tailored right ventriculotomy with patching either proximally or distally to the coronary artery and a transatrial-transpulmonary approach, with either pulmonary valvar commissurotomy or the use of a limited transannular patch [15, 16]. The choice of approach will be determined by the level of crossing at the infundibulum, the diameter of the pulmonary root and the surgeon’s experience with the various techniques [15]. The operated hearts in our archive had been historically repaired without the use of a conduit, by dissecting out the anomalous coronary arterial branch and inserting 1 or 2 infundibular patches beneath the vessel to widen the right ventricular outflow tract (Fig. 2).

The embryological development of the coronary circulation has been an area of considerable debate. Recent evidence suggests that the arterial stems grow out from the adjacent sinuses of the aortic root, rather than the arteries growing in [18]. This explains well the finding of high origin of a coronary artery from the aortic root, which can be considered a normal variant rather than a congenital anomaly (Fig. 2). We found almost one-fifth of the coronary arteries in our cohort to take their origin at or above the sinutubular junction. Such variations, nonetheless, may have implications late after surgical repair if reintervention is required because of dilation of the aortic root, which is commonly observed during adulthood [19, 20].

### Relative position of the great arteries

We found the aortic valve in its usual position, namely posterior and to the right of the pulmonary valve, in most hearts (61.9%). A side-by-side arrangement, with the aortic valve to the right of the pulmonary valve, was found in approximately a further third. This movement of the aortic valve around the pulmonary valve is significantly higher than has previously been reported, when the aorta was stated to be normally situated in up to 93% of hearts [21]. We found an association between the position of the great arteries and an aberrant coronary branch crossing the right ventricular outflow tract. In most cases where the anterior interventricular artery originated from the right coronary artery, the aorta was situated side-by-side and to the right. The only case where the aortic valve was positioned side-by-side to the left of the pulmonary valve was also the only case in which an anomalous right coronary artery crossed the subpulmonary infundibulum (Fig. 2).

### Aortic override

**Figure 7:**
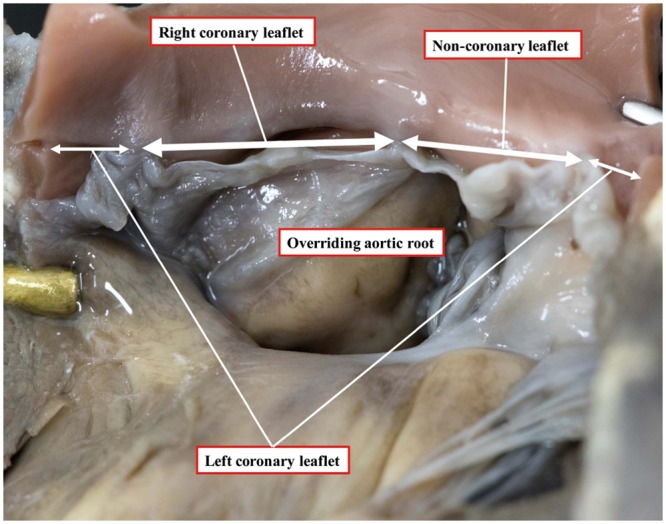
Aortic valve overriding the ventricular septal defect, with unequal leaflet sizes: the right coronary leaflet > the non-coronary leaflet > the left coronary leaflet.

The degree of aortic override has previously been assessed qualitatively by visual observation or by measuring the proportion of the aortic valvar leaflets overriding the right ventricle [13]. Both such techniques have their limitations. The qualitative method will vary depending on the angle that the observer views the aortic valve in relation to the crest of the ventricular septum and is not uniformly reproducible. Similarly, the extent of override seen on echocardiography or cross-sectional imaging will vary depending on alignment to the plane of the aortic valvar orifice. The method based on the arrangement of the aortic valvar leaflets suffers in that the leaflets themselves may not be of equal size, potentially reducing the accuracy of the method (Fig. 7). We introduced the linear method to address shortfalls in these established techniques in assessing post-mortem hearts. The technique also has potential applications in the clinical setting, with increasing use of computed datasets that permit virtual dissection. The leaflet proportion method, nonetheless, is the most useful for intraoperative assessment and provided a much more reliable approximation to the linear method than qualitative assessment.

The relationship between double-outlet right ventricle and tetralogy of Fallot has long been contentious, largely centred on the dogma that bilateral infundibulums are needed to make the diagnosis of double outlet. Other disagreements have been related to the required degree of aortic overriding, with authors recommending from more than 50% to approximately 85–90% [7, 21, 22]. Double outlet, nonetheless, is a type of ventriculo-arterial connection rather than a distinct anomaly and can be found in the setting of tetralogy [23]. In the initial description provided by Fallot himself [1], one of the hearts was described as having the aortic root exclusively supported by the right ventricle. The aortic override found in our hearts varied from 31% to 100%, including 6 hearts with the aortic root supported exclusively by the right ventricle and hence having unequivocal double-outlet ventriculo-arterial connection. This has implications for surgical repair, since in these circumstances a much larger patch will be required to baffle blood from the left ventricle and through the interventricular communication to the aortic root [24]. It follows that the area usually described as the ‘ventricular septal defect’ in the double-outlet right ventricle is never closed. It is the area patched to connect the aortic root with the left ventricle that is analogous to the ventricular septal defect as described in the setting of tetralogy of Fallot with concordant ventriculo-arterial connections.

### Ventricular septal defect

The specific morphology of the ventricular septal defect also impacts surgical decision-making. We found that a perimembranous defect was present in five-sixths of the hearts. In this arrangement, the atrioventricular conduction axis passes through the area of fibrous continuity between the tricuspid, aortic and mitral valves [25]. It is, therefore, at risk of injury during placement of the patch, with the potential for subsequent disturbances of atrioventricular conduction [4, 7]. When a muscular posterior–inferior rim is present, however, it protects the conduction axis from injury during the placement of sutures [26].

In the small number of defects that were doubly committed and juxta-arterial (1.2%), there was fibrous continuity between the leaflets of the aortic and pulmonary valves. This is the consequence of failure of formation of the muscular subpulmonary infundibulum, although there can be a fibrous outlet septum [27], and also impacts the placement of sutures. In this setting, the leaflets of the aortic valve lack support from the muscular infundibulum, with an increased risk of leaflet prolapse and aortic regurgitation. This complication may require concomitant intervention to the aortic valve to maintain or restore its integrity.

### Limitations

The hearts admitted to the archive may not be representative of the whole population of patients with tetralogy of Fallot due to selection biases, including disease severity or unsuccessful surgical repair. It was not the aim of our study, however, to estimate the prevalence of anomalies, rather to document their variability. The long-term storage of specimens in formaldehyde is recognized to lead to some degree of shrinkage; this potential caveat was countered by using ratios and relative proportions rather than absolute values. In addition, most, but not all, hearts had been dissected in a consistent way such that some hearts could not be examined for specific features due to disruption or distortion of their anatomical relationships.

## CONCLUSIONS

Anatomical variations in tetralogy of Fallot are common and may have implications for surgical management. The variability in the extent of aortic override determines the size of the patch required to baffle blood to the aorta. An anomalous coronary artery crossing the right ventricular outflow tract may require placement of a conduit rather than transannular patch. The specific morphology of the ventricular septal defect affects the site of placement of sutures during its closure and, hence, the risk of postoperative conduction defects. Access to historical cardiac archives provides a unique and valuable resource for improving our understanding of these morphological variations and to assess their impact on surgical care.

## Supplementary Material

ezy474_Supplementary_Table

## Notes

Presented at the 32nd Annual Meeting of the European Association for Cardio-Thoracic Surgery, Milan, Italy, 18–20 October 2018.
