# Supplementary material for: Tetralogy of Fallot: morphological variations and implications for surgical repair
Source: Eur J Cardiothorac Surg. 2019 Jan 16;56(1):101–9. doi: 10.1093/ejcts/ezy474 (PMC6580293; doi:10.1093/ejcts/ezy474)
Supplement: ezy474_Supplementary_Table [file ezy474_supplementary_table.pdf]

SUPPLEMENTARY MATERIAL

Table S1: Classification of observations and measurements in hearts with Tetralogy of Fallot.

**Demographic Data**		
Parameter	*Unit*	*Source*
Database Number	n	Post Mortem Report
Post Mortem Number	n	Post Mortem Report
Date of Birth	n	Post Mortem Report
Date of Death	n	Post Mortem Report
Date of Repair	n	Post Mortem Report
Gender	Male / Female / Other	Post Mortem Report
Weight	g	Post Mortem Report
Height	cm	Post Mortem Report
Head Circumference	cm	Post Mortem Report
Genetic Anomalies	free text	Post Mortem Report
Absent Thymus	n	Post Mortem Report

**General Characteristics**		
Parameter	*Unit*	*Measurement Method*
Heart Type	Naïve / Operated	Visual Inspection
Heart Shape	Boot shaped / Not boot shaped / NA	Visual Inspection
Heart Weight	g	Kern PFB Top Pan Balance
Apex Orientation	Left / Middle / Right / NA	Visual Inspection
Apex Forming Ventricle(s)	Left ventricle / Right ventricle / Right ventricle & Left ventricle / NA	Visual Inspection

Coronary Arteries		
Parameter	*Unit*	*Measurement Method*
Number of Coronary Orifice	n	Visual Inspection and Probe
Right Coronary Artery present	Yes / No / NA	Visual Inspection
Left Coronary Artery present	Yes / No / NA	Visual Inspection
Anterior Interventricular Coronary Artery arises from the Left Coronary Artery	Yes / No / NA	Visual Inspection
Anterior Interventricular Coronary Artery arises from the Right Coronary Artery	Yes / No / NA	Visual Inspection
Inferior Interventricular Coronary Artery arises from the Left Coronary Artery	Yes / No / NA	Visual Inspection
Inferior Interventricular Coronary Artery arises from the Right Coronary Artery	Yes / No / NA	Visual Inspection
Abnormal Coronary Distance	mm	String and Ruler
Anterior Interventricular Coronary Artery distance to Aorta	mm	String and Ruler
Coronary Course/Origin	Normal / Slit-like / Intramural / NA	Visual Inspection
Any other Coronary abnormalities	free text	Visual Inspection

Heart Morphology		
Parameter	*Unit*	*Measurement Method*
Atrial Arrangement	Usual Atrial Arrangement / Mirror Imagery / Left Atrial Isomerism / Right Atrial Isomerism / Juxtaposed Appendages / NA	Visual Inspection
Concordant Atrioventricular Connections	Yes / No / NA	Visual Inspection

Concordant Ventriculoarterial Connections	Yes / No / NA	Visual Inspection
Position of arterial valves<br>(pulmonary valve as reference)	Aortic Valve is Anterior / Posterior / Left / Right / Anterior & Right / Anterior & Left / Posterior & Right / Posterior & Left / NA	Visual Inspection

**Atria and Ventricles**		
Parameter	*Unit*	*Measurement Method*
Right Ventricle Inlet length	mm	Ruler
Right Ventricle Outlet length	mm	Ruler
Left Ventricle Inlet Length	mm	Ruler
Left Ventricle Outlet Length	mm	Ruler
Right Ventricle Thickness	mm	Ruler
Left Ventricle Thickness	mm	Ruler
Outlet Septum	Present / Absent / Fibrous / NA	Visual Inspection
Membranous Flap	Yes / No / NA	Visual Inspection, Probe and Illuminating Flashlight
Degree of Septoparietal Trabeculation Hypertrophy	Mild / Moderate / Severe / NA	Visual Inspection
Outlet Septum Thickness	mm	Ruler
Length of Outlet Septum	mm	Ruler
Infundibular Width	mm	String and Ruler
Total Infundibulum Length	mm	Ruler
Infundibulum Hypoplasia	Absent / Mild / Moderate / Severe / NA	Visual Inspection

**Tricuspid Valve**		
Parameter	*Unit*	*Measurement Method*

Right Atrioventricular Valve Leaflets	Trileaflet / Dysplastic / NA	Visual Inspection
Right Atrioventricular Valve Leaflets Fused	None / Anterior and Septal / Posterior and Septal / Anterior and Posterior / NA	Visual Inspection
Right Atrioventricular Valve Orifice Circumference	mm	String and Ruler
Medial Papillary Muscle Present	Yes / No / NA	Visual Inspection
Medial Papillary Muscle Origin	Posterior Limb of Septomarginal Trabeculation / Anterior Limb of Septomarginal Trabeculation / Right Ventricle Septum / NA	Visual Inspection

**Pulmonary Valve**		
Parameter	*Unit*	*Measurement Method*
Pulmonary Valve Leaflets	Trileaflet / Miniature - not dysplastic / Unileaflet / Bileaflet / Dysplastic / Stenotic / Doming / NA	Visual Inspection and Probe
Pulmonary Valve Leaflets Fused	None / Anterior and Left / Right and Left / Anterior and Right / NA	Visual Inspection and Probe
Sinotubular Junction Circumference	mm	String and Ruler
Pulmonary Valve Orifice Circumference	mm	String and Ruler
Pulmonary Nadir Circumference	mm	String and Ruler

**Mitral Valve**		
Parameter	*Unit*	*Measurement Method*
Left Atrioventricular Valve Leaflets	Trileaflet / Bileaflet / Dysplastic / NA	Visual Inspection
Left Atrioventricular Valve Orifice Circumference	mm	String and Ruler

Aortic Valve		
Parameter	*Unit*	*Measurement Method*
Aortic Valve Leaflets	Trileaflet / Bileaflet / Dysplastic / NA	Visual Inspection
Aortic Valve Leaflets Fused	None / Left Coronary Leaflet and Non-Facing Leaflet / Right Coronary Leaflet and Non-Facing Leaflet / Left Coronary Leaflet and Right Coronary Leaflet / NA	Visual Inspection
Sinotubular Junction Circumference	mm	String and Ruler
Aortic Valve Orifice Circumference	mm	String and Ruler
Sinus of Valsalva Circumference	mm	String and Ruler
Aortic Nadir Circumference	mm	String and Ruler
Aortic-Mitral Continuity	Yes / No / NA	Visual Inspection
Aortic Valve Leaflets Involved in Aortic-Mitral Continuity	Left Coronary Leaflet / Right Coronary Leaflet / Non-Facing Leaflet / Left Coronary Leaflet and Non-Facing Leaflet / Right Coronary Leaflet and Non-Facing Leaflet / Left Coronary Leaflet and Right Coronary Leaflet / Left Coronary Leaflet & Right Coronary Leaflet & Non-Facing Leaflet / NA	Visual Inspection
Aortic Override (Linear)	mm	String and Ruler
Aortic Override (Leaflets)	free text	Visual Inspection

Great Arteries – Aorta and Pulmonary Trunk		
Parameter	*Unit*	*Measurement Method*
Ascending Aorta Circumference	mm	String and Ruler
Aortic Arch	Left / Right / Double / NA	Visual Inspection
Aortic Coarctation	Yes / No / NA	Visual Inspection

Aortic Arch Hypoplasia	Proximal / Distal / Isthmus / None / NA	Visual Inspection
Aortic Branching	Normal / Mirror / Abnormal / Free Text / NA	Visual Inspection
Pulmonary Trunk circumference	mm	String and Ruler
Number of Pulmonary Arteries	Two / NA	Visual Inspection
Angle Between Main Pulmonary Artery and Right Pulmonary Artery	Degrees / NA	Protractor
Angle between Main Pulmonary Artery and Left Pulmonary Artery	Degrees / NA	Protractor
Right Pulmonary Artery Circumference	mm	String and Ruler
Left Pulmonary Artery Circumference	mm	String and Ruler

**Septal Defects**		
Parameter	*Unit*	*Measurement Method*
Atrial Septum	Intact / Atrial Septal Defect / Patent Foramen Ovale / NA	Visual Inspection and Probe
Ventricle Septal Defect	Muscular Posterior Inferior Rim / Muscular Outlet / Perimembranous / Doubly Committed / Other / NA	Visual Inspection and Torch
Ventricle Septal Defect Circumference	mm	String and Ruler
Conduction Axis visible on Left Ventricle Septum	Yes / No / NA	Visual Inspection and Torch
Atrioventricular Septal Defect	Yes / No	Visual Inspection

**Other Anomalies**		
Parameter	*Unit*	*Measurement Method*
Left Superior Vena Cava Present	Yes / No / NA	Visual Inspection and Probe
Arterial Duct	Patent / Closed / Absent /	Visual Inspection and

	Right / NA	Probe

Operated Hearts		
Parameter	*Unit*	*Measurement Method*
Shunt	Yes / No / NA	Visual Inspection and Probe
Surgical Incision Type	Atriotomy / Ventriculotomy / Both / NA	Visual Inspection
Length of Incision from Base of Right Superior Vena Cava to Inferior Limit of Incision	mm	String and ruler
Length of Incision from Ventriculoarterial Junction to Inferior Limit of Incision	mm	String and ruler
Transannular Patch	Yes / No / NA	Visual Inspection
Transannular Patch Material	Pericardium / Dacron / NA	Visual Inspection
Right Ventricle-Pulmonary Artery Conduit	Yes / No / NA	Visual Inspection
Main Pulmonary Artery Patch	Yes / No / NA	Visual Inspection
Right Pulmonary Artery Patch	Yes / No / NA	Visual Inspection
Left Pulmonary Artery Patch	Yes / No / NA	Visual Inspection
Ventricle Septal Defect Patch	Pericardium / Dacron / NA	Visual Inspection
Ventricle Septal Defect Patch Circumference	mm	String and ruler
Other Procedures	free text	Visual Inspection

Table S2: Methodology for measurements in hearts with Tetralogy of Fallot.

Parameter	*Description of Measurement*
Heart Weight	Hearts were weighed at the end of recording all observations/measurements to give maximal time for formalin to evaporate so it does not overestimate the heart weight
Abnormal Coronary Distance	Distance from mid-point of the right ventriculoarterial junction perpendicularly down to any coronary artery or coronary branch passing across the right ventricle outflow tract
Distance between the Anterior Interventricular Coronary Artery and Aorta	Shortest distance measured from the aorta along the right ventriculoarterial junction to the anterior interventricular coronary artery
Right Ventricle Inlet length	Shortest distance from the tricuspid valve annulus to the right ventricle apex
Right Ventricle Outlet length	Shortest distance from the pulmonary valve base/nadir to the right ventricle apex
Left Ventricle Inlet Length	Shortest distance from the mitral valve annulus to the left ventricle apex
Left Ventricle Outlet Length	Shortest distance from the aortic valve base/nadir to the left ventricle apex
Right Ventricle Wall Thickness	Maximum wall thickness excluding trabeculations
Left Ventricle Wall Thickness	
Outlet Septum Thickness	Maximum thickness
Outlet Septum Length	Shortest distance from the pulmonary valve base/nadir to the edge of the outlet septum
Infundibular Width	Narrowest internal circumference of sub-pulmonary infundibulum
Right Atrioventricular Valve Orifice Circumference	Internal circumference
Pulmonary Sinotubular Junction Circumference	
Pulmonary Valve Orifice Circumference	
Pulmonary Nadir/Base Circumference	

Left Atrioventricular Valve Orifice Circumference	Internal circumference
Aortic Sinotubular Junction Circumference	
Aortic Valve Orifice Circumference	
Sinus of Valsalva Circumference	
Aortic Nadir/Base Circumference	
Aortic Override (Linear)	(Aortic valve nadir circumference – Aortic valve nadir circumference that lies in the left ventricle) / Aortic nadir circumference
Ascending Aorta Circumference	Internal circumference
Pulmonary Trunk circumference	
Right Pulmonary Artery Circumference	
Left Pulmonary Artery Circumference	
Ventricle Septal Defect (VSD) Circumference	Measurement of circumference from the right ventricle
Length of Incision from Base of Right Superior Caval Vein to Inferior Limit of Incision	External measurement made with the heart in the anatomical position to have the superior caval vein base in the plane similar to when it is in situ
Length of Incision from Ventriculoarterial Junction to Inferior Limit of Incision	
Ventricle Septal Defect Patch Circumference	Used as a surrogate to measure the actual size of VSD. The circumference measured was within the VSD patch suture line rather than outside as sutures are placed at the VSD edge and the actual VSD lies within the area of the sutures placed
